# A New Automated Way to Measure Polyethylene Wear in THA Using a High Resolution CT Scanner: Method and Analysis

**DOI:** 10.1155/2014/528407

**Published:** 2014-01-22

**Authors:** Gerald Q. Maguire Jr., Marilyn E. Noz, Henrik Olivecrona, Michael P. Zeleznik, Lars Weidenhielm

**Affiliations:** ^1^School of Information and Communication Technology, KTH Royal Institute of Technology, Isafjordsgatan 26, 418 164-40 Stockholm, Sweden; ^2^Department of Radiology, New York University, 550 First Avenue TSHW232, New York, NY 10016, USA; ^3^Department of Molecular Medicine and Surgery, Section of Orthopaedics and Sports Medicine, Karolinska Institute, A2:07, 171 76 Stockholm, Sweden; ^4^School of Computing, University of Utah, College of Engineering, 50 Central Campus Dr., Room 3190, Salt Lake City, UT 84112, USA

## Abstract

As the most advantageous total hip arthroplasty (THA) operation is the first, timely replacement of only the liner is socially and economically important because the utilization of THA is increasing as younger and more active patients are receiving implants and they are living longer. Automatic algorithms were developed to infer liner wear by estimating the separation between the acetabular cup and femoral component head given a computed tomography (CT) volume. Two series of CT volumes of a hip phantom were acquired with the femoral component head placed at 14 different positions relative to the acetabular cup. The mean and standard deviation (SD) of the diameter of the acetabular cup and femoral component head, in addition to the range of error in the expected wear values and the repeatability of all the measurements, were calculated. The algorithms resulted in a mean (±SD) for the diameter of the acetabular cup of 54.21 (±0.011) mm and for the femoral component head of 22.09 (±0.02) mm. The wear error was ±0.1 mm and the repeatability was 0.077 mm. This approach is applicable clinically as it utilizes readily available computed tomography imaging systems and requires only five minutes of human interaction.

## 1. Introduction

Total hip arthroplasty (THA) devices are being utilized for longer periods of time as younger and more active patients receive them [1]. Although there are a variety of common reasons for long-term failure [2, 3], this study concerns only wear [4]. *In vivo* wear rates of several different acetabular cups, with and without polyethylene liners, have been reported [5–13] with the most recent liner wear rates ranging from 0.037 mm/year to 0.005 mm/year and total wear at revision being about 1.0 to 3.5 mm. Higher precision and accuracy of wear assessment methods would shorten the time for clinical studies of new implants and enable detection of clinically significant wear [14].

Previously our group showed that CT volumes can be used to evaluate acetabular cup position and migration in hip phantoms and patients [15, 16] and to determine 3D migration of the femoral component head into the acetabular cup at 1 mm, later reduced to 0.51 mm [17]. However, this required considerable user interaction time as about 200 landmarks (points) had to be placed on the 3D surfaces of the femoral component head and acetabular cup.

Here the interaction time to choose landmarks is reduced by limiting the number of landmarks to a total of seven (requiring only five minutes on average per CT volume) and the skill level of the operator was reduced. Surfaces were automatically extracted based on these landmarks plus a threshold for the electron density of the prosthetic material; the center and diameter of the prosthetic components viewed as (parts of) spheres were automatically determined, and from these centers, the distance between the acetabular cup and femoral component head is inferred. The entire process takes roughly 20 minutes.

## 2. Methods and Materials

A hip phantom, used to simulate the 3D displacement of the femoral component (CoCr PROTASUL-20, Zimmer Inc., Warsaw, ID) toward the acetabular cup (Inter-OpTM, Zimmer Inc., Warsaw, ID, formally Sulzer Orthopaedics), was scanned in a high resolution prototype flat panel CT scanner (Siemens Medical Solutions, Erlangen, Germany) [18]. The same experimenter acquired two series of 14 scans separated by five days. Before each scan, the hip phantom was removed from the CT scanner, the micrometer was set as indicated in Table 1, and then the hip phantom was replaced with the femur roughly aligned with the *z*-axis of the scanner. Each scan was identified by a random letter. The CT scan parameters were 100 kVp and 50 mA, with a spatial resolution of 200 *μ*m on a side, a matrix size of 512 × 512, and 390 slices for the first and 385 slices for the second scan.

The phantom was disassembled and the diameter of the acetabular cup was measured using a coordinate measuring machine (CMM) (Global Advantage, Brown and Sharp, North Kingstown, RI) with special software (PC-DMIS, Version 1.0, Wilcox Associates, Inc., North Kingstown, RI) by an outside vendor (Hexagon Metrology, Inc., North Kingstown, RI). The ruby probe tip had a diameter of 3 mm on a 20 mm shaft. A total of 8 probe positions (each with an accuracy of 0.000254 mm and an assumed repeatability of 0.000508 mm) were used. The diameter of the femoral component head was measured using a caliper (Mitutoyo Corporation Digimatic, Toronto, ON, Canada), calibrated by the manufacturer. The same experimenter made 10 measurements moving the caliper to a different position each time.

A 3D image processing tool, previously described in [19, 20], was used to choose seven landmarks, readily identifiable in a patient. These landmarks were used only to limit the CT data volume searched to find the surfaces of the acetabular cup and the femoral component head. Details of the hardware and software are given in the appendix.

### 2.1. Procedure for Choosing Landmarks and Algorithm for Automatic Surface Extraction

**Figure 1:**
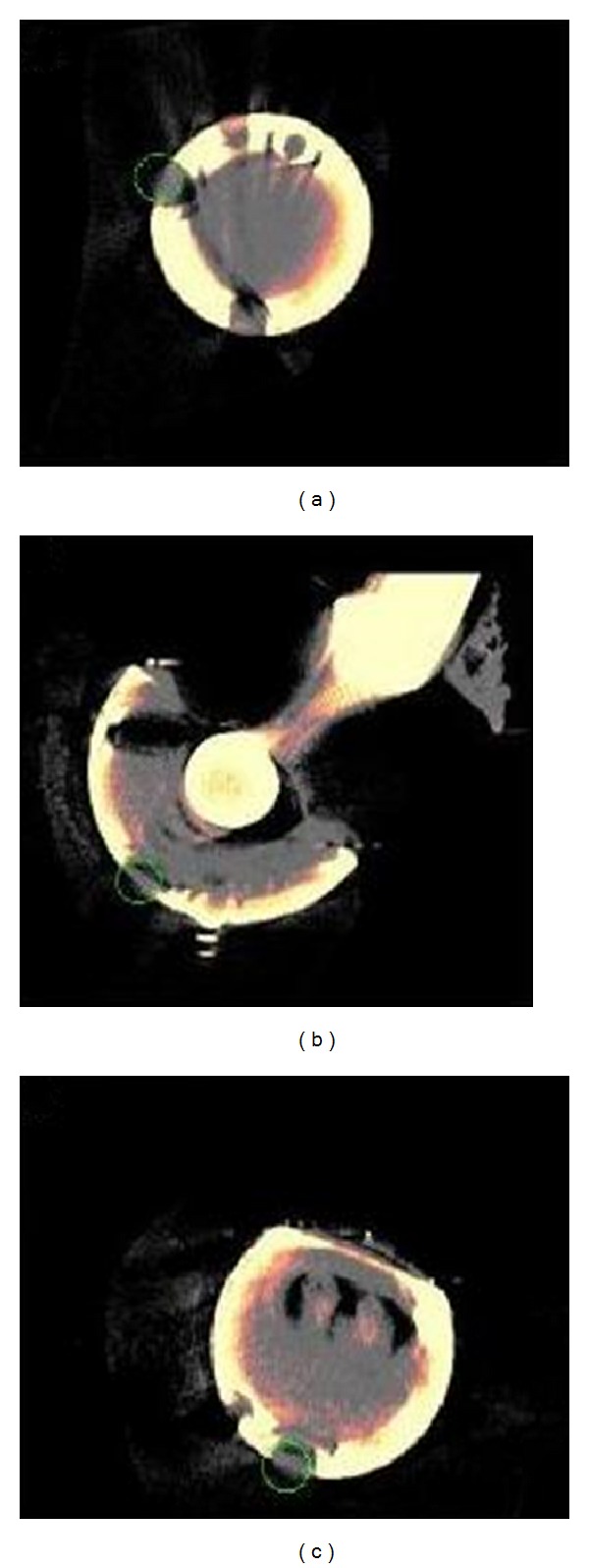
Three 2D orthogonal projections showing the 3D spherical landmark located in the apical dome hole. (a) shows the axial projection, (b) the coronal, and (c) the sagittal.

**Figure 2:**
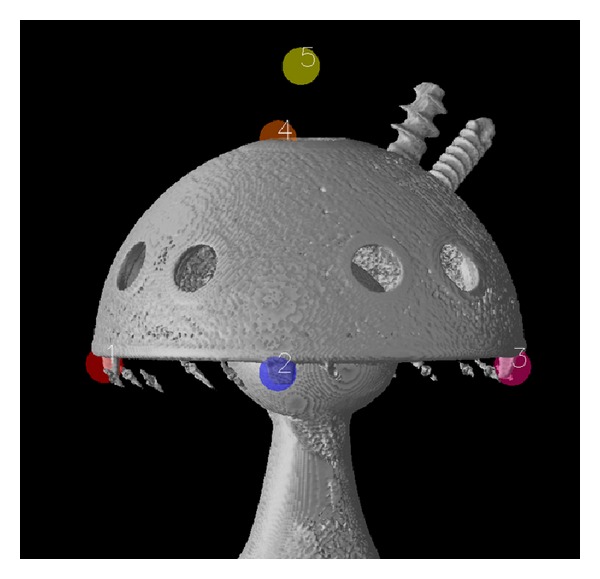
The five landmarks chosen on the acetabular cup are shown in 3D. Only landmarks 1, 2, 3, and 5 are used in the surface extractor calculations. Landmark 4 is used only by the operator to locate the apical dome hole.

**Figure 3:**
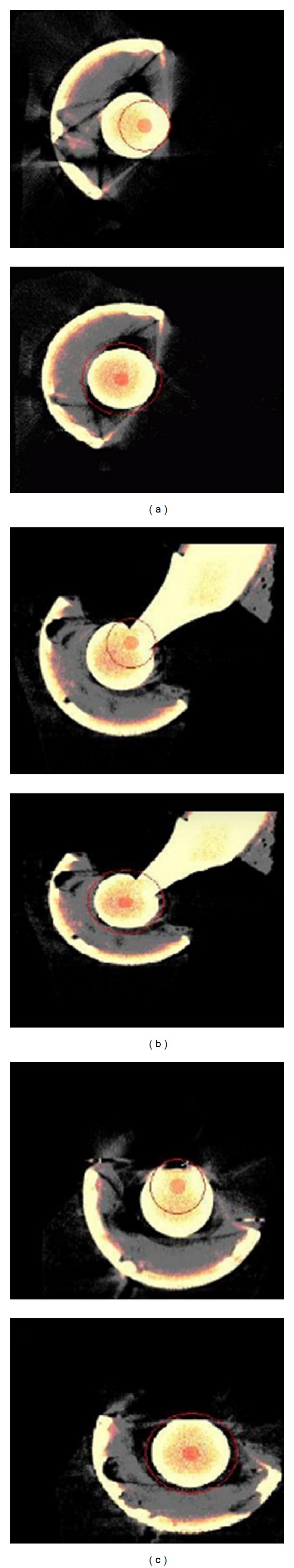
The top row contains the three 2D orthogonal projections showing the 3D spherical landmark located at the junction between the femoral component head and stem and the bottom row contains the three 2D orthogonal projections showing the 3D spherical landmark surrounding the femoral component head. (a) shows the axial projection, (b) the coronal, and (c) the sagittal.

(A) Viewing each CT volume as a 3D isosurface, the observer placed a landmark on three noncolinear points on the bottom of the acetabular cup to define a plane. A fourth landmark was placed near the apex (apical dome hole). Viewing the CT volume as 2D orthogonal projections: transverse, coronal, and sagittal (Figure 1), the observer centered a suitably sized 3D sphere in the apical dome hole and on the coronal projection, shifted it diagonally out a short distance (5 to 10 mm), and created a landmark (Figure 2).

(B) A surface extraction algorithm used these landmarks and a threshold (based on the acetabular cup material and the peak kilovoltage) to limit the search. The shortest distance between the defined plane and the point centered in the apical dome hole defined a starting radius. The software searched from the outside and ranged for only 60% of the starting radius to eliminate unused screw holes.

(C) Using 2D orthogonal projections the observer interactively placed a 3D sphere so as to surround the junction between the femoral component head and stem and placed a landmark. A new 3D sphere was then placed which encompassed the femoral component head and a landmark was placed. This landmark together with the stem landmark constrained the search for the surface from extending into the stem (Figure 3).

(D) The surface extraction algorithm searched from the outside of the femoral component head inward and ended when the voxel value no longer exceeded the threshold, as femoral component heads are not necessarily composed of solid metal.

(E) For either surface, the algorithm found points where the voxel values along the search path consistently exceeded the threshold so that isolated voxels (above threshold) are ignored.

### 2.2. Algorithm for Automatic Sphere Fitting to the Extracted Surfaces

The surface extraction algorithm produced a set of initial landmarks (2,000–16,000) for each surface and the sphere fitting algorithm used these to estimate the actual center of the hemisphere or sphere (as appropriate) and to compute the diameter as these landmarks must statistically be consistent with a given center and diameter. The sphere fitting algorithm selected 80,000 random sets (*x*, *y*, *z*) of four surface landmarks (with every pair separated by at least 2 mm to avoid degenerate cases) to estimate a radius and center. The first time, a 1000 bin histogram of possible integer radii was computed and the maximum bin defined the initial radius value. Next, this procedure was repeated and statistics were kept per landmark of how many times that landmark contributed to an estimate of a radius that exceeded the initial radius value by ±2 mm. Landmarks with high values (greater than 1,600) were removed. This was repeated again and statistics were kept per landmark of how many times the landmark contributed to the estimate of a radius that exceeded the initial radius value by twice the slice thickness (±0.4 mm). Typically the initial set of landmarks, approximately 15,000 for the acetabular cup and 2,000 landmarks for the femoral component head, was reduced to 12,500–13,000 and 950–1,100, respectively.

### 2.3. Observer Interaction

The initial seven landmarks were chosen by the same observer (who was not the experimenter and had no knowledge of the scan order) on scan series one on one day and again nine days later. The observer chose landmarks on scan series two one day after those for scan series one and again twelve days later.

### 2.4. Analysis

For each scan, after landmarks selection, the surface extraction algorithm and the sphere fitting algorithm were applied. The initial and final landmarks were visually verified for correct placement on the intended surface. The calculated separation (CupHeadSeparation) was simply the distance between the defined sphere centers. All data, including the scan identifier, initial radius and center (*x*, *y*, *z*), and the final (best) radius and center (*x*, *y*, *z*) all in millimeters, were saved in a file as a comma separated list (CSV).

### 2.5. Statistical Analysis

For each trial, the sphere diameters were tabulated, resulting in a total of 56 data points (2 trials ∗ 14 scans ∗ 2 series) for the acetabular cup and for the femoral component head. These diameter data were tested graphically for normality (histogram, box, density, and quantile-quantile plots) and then the median, mean, standard deviation (SD), and coefficient of variance were calculated, as well as the 99% confidence interval (CI). For each trial of each scan, the CupHeadSeparation was calculated and the difference from the expected value based on the micrometer settings (Table 1) was calculated and plotted. The repeatability of the test results was evaluated as outlined in [21, 22] as well as the 99% CI. The repeatability was defined as the precision under conditions where independent tests are conducted with the same method on the same test item in the same laboratory by the same observer using the same equipment, within a short interval of time. R version 2.11.1 was used for all statistical calculations.

## 3. Results

**Figure 4:**
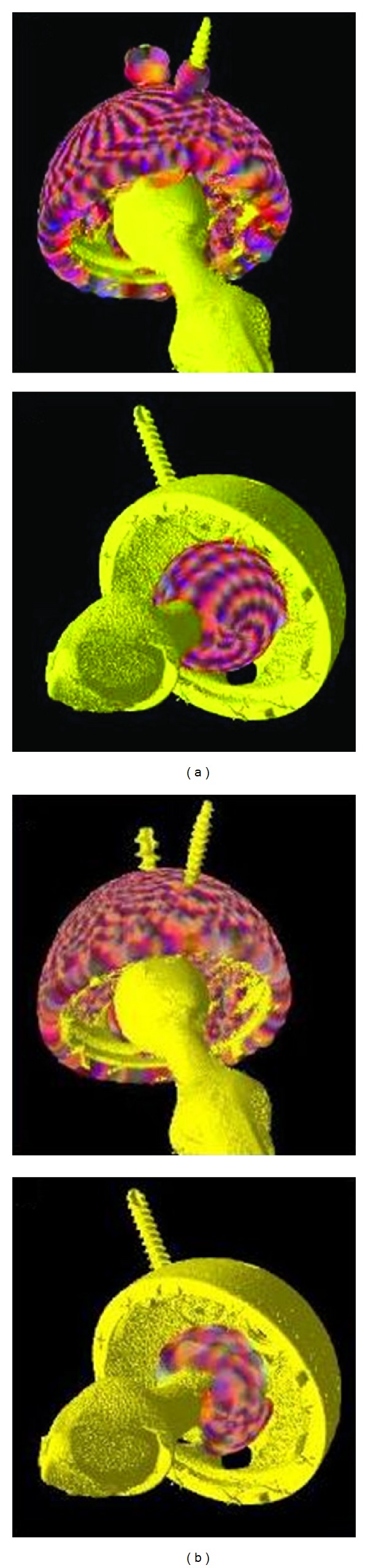
The top row illustrates the initial and valid surface landmarks produced on the acetabular cup by the surface extraction algorithm and the bottom row shows those for the femoral component head. The set of initial landmarks produced is shown in (a). The set of valid landmarks is shown in (b). The overlapping colored spherical glyphs represent the generated landmarks.

All scans in both series were able to be analyzed for both trials. The visual check of the generated surface landmarks after surface extraction (Figure 4(a)) and again after sphere fitting (Figure 4(b)) confirmed that the landmarks were correctly placed for the acetabular cup (top row) and the femoral component head (bottom row).

**Figure 5:**
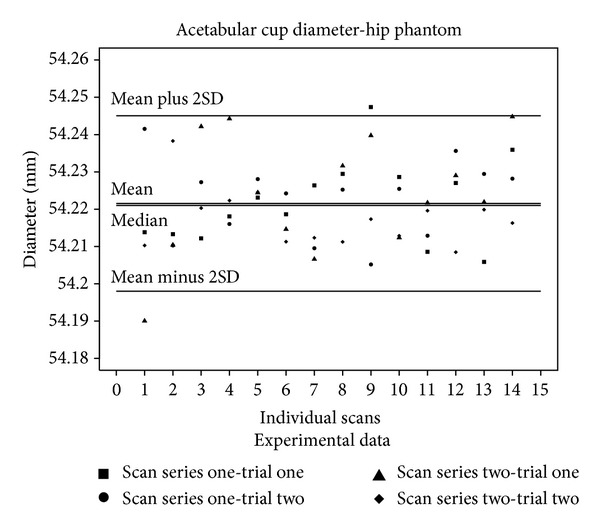
The values of the acetabular cup diameters obtained from each individual calculation are shown with the median, mean, and two SD lines drawn through the data. The median value is quite close to the mean value.

**Figure 6:**
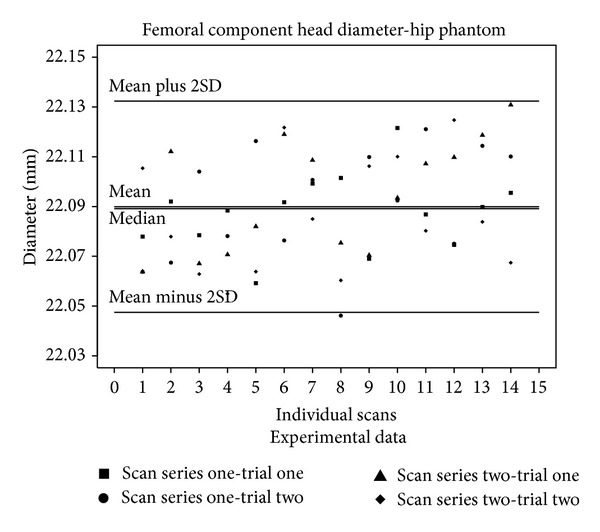
The values of the femoral component head diameters obtained from each individual calculation are shown with the median, mean, and two SD lines drawn through the data. The median value is quite close to the mean value.

The calculated diameter for both the acetabular cup and the femoral component head was graphically checked to confirm that the data were normally distributed. The diameter mean and median values, which were almost equal, for the acetabular cup and femoral component as well the coefficient of variance, the 99% confidence interval, and values obtained by the CMM and caliper measurements are given in Table 2.  Figure 5 illustrates the acetabular cup data graphically and Figure 6 does the same for the femoral component head.

**Figure 7:**
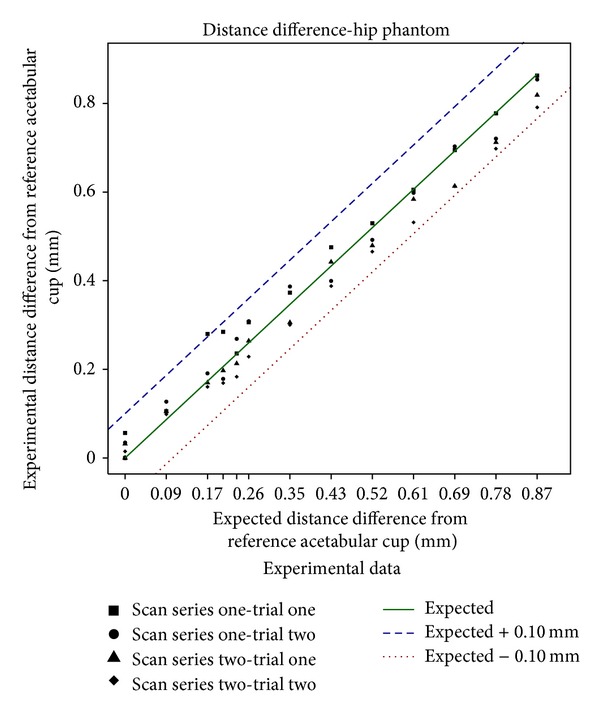
The calculated CupHeadSeparation plotted against the expected CupHeadSeparation from Table 1, for scan series one and two (both trials). Lines are drawn at the exact expected value and at ±0.1 mm demonstrating the data were within 0.1 mm of the expected distance.

For each trial of each scan, the CupHeadSeparation was calculated along with the difference between this and the expected CupHeadSeparation based on the micrometer settings (Table 1). The reference scan was the first scan in which each micrometer was set to zero. For both scan series and trials, the linear least squares line fit between the measured and expected values was quite close to a 45° line; that is, the measured and expected values were approximately equal (Figure 7). The residuals of the regression, the line intercept, and the slope values are summarized in Table 3. As can be seen from Figure 7, all the distance results fell within the interval of ±0.1 mm with a mean of −0.013 mm, a SD of ±0.038 mm, and a 99% CI of −0.031 to 0.006 mm. Repeatability was 0.077 mm for the 224 (14 scans ∗ 4 (radius + center points) ∗ 2 trials ∗ 2 series) values. The mean and median of the difference in each value were 0.004 mm and 0.003 mm, respectively, with a standard deviation of ±0.028 mm and a 99% CI of 0.009 to −0.0004 mm.

## 4. Discussion

Existing wear measurement techniques range from simple single radiographic techniques to more advanced three-dimensional (3D) techniques [4, 5, 23–30]. The most accurate 3D wear monitoring method today is radiostereometric analysis (RSA) [31, 32]. However, RSA is normally not available in clinical practice since it requires special stereotactic X-ray equipment and implantation of small tantalum balls. For 3D wear assessment current multislice computed tomography (CT) offers accurate spatial volume resolution without significant metal artefacts, now well suppressed by the CT manufacturers' software. A measurement of femoral component head penetration into polyethylene using a 3D CT technique was reported in [23].

The present study reports a method to assess polyethylene wear by measuring the displacement of the femoral component head relative to the acetabular cup, thus giving a baseline for an ideal situation. In a clinical situation, there is the possibility that the femoral component head is not seated at the most worn part of the polyethylene, especially in the presence of multiple wear tracts as demonstrated in our previous study [33]; there is a risk of distortion of the volume due to patient movement during the scan, and finally, the proposed method was devised for uncemented acetabular components of hemispherical configuration which comprise the majority of acetabular cups in clinical use today. The agreement of ±0.1 mm between the calculated CupHeadSeparation and the expected CupHeadSeparation was reasonably good for both scan series and suggests that this method could be used clinially to detect differences of greater than 0.1 mm. The centers of the acetabular cup and the femoral component head are implicitly known to be within ±1 mm (SD ± 0.013 mm and ±0.03 mm, resp.). Hence the distance between the centers should be within ±0.086 mm of the actual distance. This improves the previous results from our group [17] but is about half the accuracy of phantom study results presented in [23]. However, this new method does not need special reconstruction of the raw CT data and the computer aided design (and manufacturing) data for the actebular cup and femoral component, nor does it need 40 points on the femoral component head and 20 or more around the bottom rim of the acetabular cup. The software for the method presented is system independent whereas [23] uses the software package OSIRIS [34] which runs only on an Apple computer. The accuracy and repeatability in [23] were determined by repeated measurements on a calibrated 28 mm prosthetic head and by comparing them with direct metrological measurements on acetabular specimens with *in vitro* wear from machining and on explanted acetabular specimens with *in vivo* wear. Estimated femoral component penetration in both all-poly and metal-backed acetabular components ranged from 0.009 to 0.245 mm with a mean of 0.080 mm and SD 0.067 mm [23].

The calculated acetabular cup diameter measurements in this study were approximately 0.6 mm less than that obtained by CMM because the CMM probe tip (3 mm) was too large to penetrate the projections of the rough trabecular metal mesh surface of the acetabular cup, resulting in a larger diameter defined by the highest projections of this surface. However, our sphere fitting function utilizes CT voxels representing the full thickness of the metal surface without the 0.3 mm thick trabecular coating. The estimated CT measurements of the femoral component head diameter were very close to the caliper measurement. These diameter measurements provide an indirect indication of the accuracy and repeatability of finding these surfaces. An additional aspect of this method is that with the addition of one more landmark on the acetabular cup there is sufficient data to register the same acetabular cup in two different CT volumes taken at different times.

The particular implant used here was discontinued due to a manufacturing error and the CT scanner was a prototype. Therefore, after the experiments described above we repeated the setup using a presently available acetabular cup (Cluster Holed Trilogy cup, diffusion bonded to a Tivanium TI-6AL-4V alloy, Zimmer Inc., Warsaw, ID) with a nominal diameter of 56 mm. The femoral component (Zimmer Inc., Warsaw, ID) had a nominal head diameter of 32 mm. We scanned this phantom on a commercially available clinical CT scanner (SOMATOM Definition Flash, Siemens Medical Solutions, Forcheim, Germany) which has an *x*-*y* pixel size of 0.35 mm and a slice thickness of 0.6 mm. The volumes for this scan series were acquired with a matrix size of 512 × 512 for 357 slices. Preliminary analysis gave a mean (SD) for acetabular cup diameter and femoral component head, respectively, of 54.85 (±0.03) mm and 32.23 (±0.07) mm. As described by Goldvasser et al. [35], the method has been shown to accurately estimate the displacement of the acetabular cup diameter and femoral component head: *x*-axis 0 mm (SD 0.213), *y*-axis 0.039 mm (SD 0.035), and *z*-axis 0.039 mm (SD 0.051). Although it was possible to resolve the CupHeadSeparation to ±0.1 mm, this needs further confirmation. We are currently calculating the liner wear from preoperative CT scans of patients who had their prosthesis explanted. These results are being compared to those obtained from direct measurement of the liner using calibers and CMM. On 11 samples thus far the results are consistent with those presented here. Future work will investigate simplification of this method as clinically, only the distance between the centers need be determined. Hence it should be possible to reduce the number of landmarks from seven to three: one to indicate where the stem of the femoral component joins the head, one spherical landmark that encloses the femoral component head, and one spherical landmark that encloses the acetabular cup. In this approach the surface of the femoral component head would first be found (establishing its radius and center), then the algorithm could search outward to find the outer acetabular cup surface. The third spherical landmark would limit this search. The outer surface of the acetabular cup would then be used to find the center of the acetabular cup, hence the distance between the centers can be computed. Note that this method would not be suitable if the femoral component is in direct contact with the acetabular cup.

## 5. Conclusions

In this paper we presented a potentially clinically applicable method to determine polyethylene wear associated with THA. This is important because younger and more active people are receiving these implants and they are living longer. As the first operation is the most successful one, if the liner only could be replaced, the patient would be spared a more extensive operation. The ability to replace the liner depends on early detection of liner wear. This method utilizing widely available CT machines brings us closer to this goal of timely detection of liner wear.

## Figures and Tables

**Table 1:** Phantom displacement and distance difference as determined from the micrometer settings.

Scan series identifier (letters identify scan 1 to 14 in each series)		Displacement (mm)			Expected CupHeadSeparation (mm) distance between acetabular cup and femoral component head centers relative to the reference scans (T, A) with a starting position of 0.00 mm
One	Two	*X*	*Y*	*Z*	
T	A	0.000	0.000	0.000	0.000
K	G	0.000	0.000	0.000	0.000
X	T	0.050	0.050	0.050	0.087
W	F	0.100	0.100	0.100	0.173
N	R	0.150	0.100	0.100	0.206
P	M	0.150	0.150	0.100	0.235
A	Y	0.150	0.150	0.150	0.260
R	D	0.200	0.200	0.200	0.346
H	W	0.250	0.250	0.250	0.433
Z	C	0.300	0.300	0.300	0.520
D	K	0.350	0.350	0.350	0.606
M	P	0.400	0.400	0.400	0.692
E	V	0.450	0.450	0.450	0.779
S	Z	0.500	0.500	0.500	0.866

**Table 2:** Acetabular cup and head diameter measurements (the mean and median were quite close) based on experimental results and physical measurements.

Component	Diameter (mm)	Coefficient of variation	99% Confidence interval (mm)		Measured (mm)
	Mean (±SD)		Lower	Upper	
Acetabular cup	54.22 (±0.011)	0.024	54.226	54.234	54.855^a^
Femoral head	22.09 (±0.02)	0.14	22.09	22.11	22.10 (±0.01)^b^

^a^CCM, ^b^Calipers.

**Table 3:** Linear regression analysis results based on 45° line for experimentally determined distance versus expected measurements.

	Value	Standard error	*P* value
Regression residuals	3208.22 (*F*-value)	0.02	0
Line intercept	0.02	0.01	0.01
Slope	0.95	0.02	0

## Appendix

### Hardware and Software Specifications

The programs to calculate the sphere diameters and centers were written completely in C with no special libraries other than the libc and libm required and are compiled under standard GCC. The programs run on any standard Linux/UNIX configuration (we tested on SuSE 10.3, 11.x, and 12.x) and any standard PC (we tested on two different computers equipped with an Intel Xenon CPU one at 3.60 GHz and the other at 2.80 GHz (with the software and OS in 32 bit mode on both machines), two different Intel Pentium 4 CPUs each at 3.0 GHz (in 32 bit mode), an Intel Core 2 Duo CPU E6850 at 3.0 GHz (64 bit mode), and an Intel Core 2 Quad CPU Q9550 at 2.83 GHz (in 64 bit mode), an Intel Pentium D CPU at 2.80 GHz (in 64 bit mode), and an Intel i7 950 CPU at 3.07 GHz) with configurations of 4–16 GB of memory. The running time for the extraction of the acetabular cup and femoral component head surface was 3–5 seconds, whereas the time for calculating the sphere center is 2 to 7 minutes, depending on the memory available and the speed of the CPU. The code used only a single processor; that is, it was single threaded code.
